# Acknowledgment to Reviewers of *Life* in 2020

**DOI:** 10.3390/life11020073

**Published:** 2021-01-20

**Authors:** Life Editorial Office

**Affiliations:** MDPI AG, St. Alban-Anlage 66, 4052 Basel, Switzerland

Peer review is the driving force of journal development, and reviewers are gatekeepers who ensure that *Life* maintains its standards for the high quality of its published papers. Thanks to the cooperation of our reviewers, in 2020, the median time to first decision was 16 days and the median time to publication was 35 days. The editors would like to express their sincere gratitude to the following reviewers for their precious time and dedication, regardless of whether the papers were finally published:
Abbasi, NaghmehLi, HeAbbey, DeeptiLi, Po-HsienAbbot, DorianLi, SimengAbyzov, AntonLi, SinanAccardi, LuisaLiberale, LucaAci-Sèche, SamiaLibra, MassimoAdams, SolomonLiehr, ThomasAdsuar, José CarmeloLiguori, GiovannaAfonso, Clélia NevesLih-Ming, YiinAguado, JulioLim,Chin, YanAi, YongLin, Chien-yuAine, MattiasLin, Dar-ShongAirapetian, VladimirLin, Ya-wenAlberta, LuccheseLindensmith, ChristianAleksandrovych, VeronikaLindkvist, KarinAleksandrzak-Piekarczyk, TamaraLingam, ManasviAlex, AnoopLink-Lenczowski, PawełAlexandrov, Oleg S.Lippert, Rachel N.Alexeyev, MikhailLitwin, TomaszAlfonso, SébastienLiu, HongweiAl-Mutairi, NawafLiu, Yu-HueiAltwegg, KathrinLizarraga, Karlo J.Alvarenga, LunaLlorente, BriardoAlves, C. HenriqueLodi, AlessiaAmarelli, CristianoLöllgen, HerbertAmbrosini, GraziaLonardo, AmedeoAmbrus, AttilaLongo, AntonioAmeta, SandeepLopes Torres, Eduardo JoséAnand, KartikLopes-Marques, MónicaAnand, RuchikaLopes-Ramos, Camila M.Andersen, Heidi LieLópez Fernández, HugoAndreasen, RasmusLopez-Escamez, Jose A.Andréasson, ClaesLópez-Sanmartín, MonserratAndree, Karl B.Los, DmitryAndreea, LupituLowin, TorstenAng, Pei SanLozano-Vidal, NoeliaAngelidi, AngelikiLu, Chi-JieAngelova, Plamena R.Luddi, AliceAnsari, Mohammad YunusLuijkx, KatrienAnselmi, MassimilianoŁukomska, AgnieszkaAppel, JensLukov, GeorgiArgenta, PeterLukowitz, WolfgangArianna, BellucciLuo, JinghuiArimura, ShinichiLvov, AndreyArmentano, IlariaLymberopoulos, AristotelisArnaiz, AnaMa, Chih-MingArora, Arinder K.Macek, MilanArora, GunjanMacKerell, AlexanderAshihara, EishiMadalina, GeorgescuAshraf, Ghulam MdMaddelein, Marie-LiseAttanayake, Renuka N.Madonov, PavelAtzori, LauraMagdinier, FrederiqueAudrito, ValentinaMagnuson, AnnAveic, SanjaMaj, MalgorzataAvolio, ElisaMakarenkov, VladimirB. Brandt, EricMalachowska, BeataBabatunde, KehindeMalec, PrzemysławBadawy, Amro ElMalewski, TadeuszBaier, ThomasMallard, Alistair R.Bajek, AnnaMallipeddi, SrikrishnanBalan, ShabeeshMallm, Jan PhilippBalaphas, AlexandreMallo, NataliaBalbi, AmedeoMally, FranziskaBalčiauskienė, LaimaMalvankar, Nikhil S.Balduini, AlessandraMalysh, Julia M.Balieva, Flora N.Mancini, EmilianoBaltar, FedericoMandelbaum, DavidBanerjee, SudipMangiaterra, GianmarcoBankoti, KamakshiMaqoud, FatimaBanožić, MarijaMárialigeti, KárolyBaqué, MickaelMarie-Catherine, SfornaBarabas, PeterMarnocha, CassandraBarashkov, Nikolay A.Marquardt, SebastianBarberio, BrigidaMarsh, Michael H.BARGELLINI, AnnalisaMarshall, Sarah A.Barillot, RomainMartí, EvaBarna, BalazsMartic, Tamara NikusevaBarone, GiulioMartín San Agustín, RodrigoBaroni, StefanoMartin, CesarBartel, SabineMartinelli, MariannaBartels, TimMartini, PaoloBartlett, StuartMartino, SabataBartnik, EwaMasalova, Olga V.Bartoszewski, RafalMasarone, DanieleBasak, ShibajiMassa, ChiaraBaselet, BjornMasson, Glenn R.Bashan, AnatMasuda, MariBastianini, MariaMasuda, TetsuyaBatista, RamonMaterniak-Kornas, MagdalenaBattistelli, MichelaMáthé, CsabaBautista, RocíoMathioudakis, Alexander G.Baxter, EvaMatsubara, KeikoBaydur, AhmetMatsuda, YokoBecciolini, AndreaMatsuya, YusukeBecker, SidneyMatsuzaki, ToshiyukiBedini, GianniMattes, KlausBehar, AlbertoMatulic, MajaBehr, MatthiasMatusik, PawelBeijers, LianMauricio, Maria DoloresBeit-Yannai, ElieMawardi, HaniBekri, SoumeyaMazur, ArthurBelguesmia, YanathMazur-Bialy, AgnieszkaBella, Claudia DiMcGhie, DavidBellucci, LucaMcKendry, JamesBelluzzi, ElisaMcKenzie, FredericBel’skaya, LyudmilaMcLay, ToddBenabdellah, KarimMcMenamin, MarkBenjamin, CressiotMehta, SanjayBennardo, LuigiMei, FengBennett, Annemarie E.Meiring, Rebecca MaryBerezin, AlexanderMeka, Durga PraveenBergougnoux, AnneMelekhina, Elena N.Berlec, AlešMellies, JayBernacchia, GiovanniMemo, MaurizioBernasconi, CorradoMena, SalvadorBertoglio, DanieleMénard, CarolineBertolet, AlejandroMercedes, GimenoBestwick, MeganMercier, CorinneBette, MichaelMeriggi, PaoloBetzer, CristineMerigo, FlaviaBhaskara, Govinal BadigerMerkely, BélaBhati, Kaushal KumarMešić, ArminBiagi, MarcoMesquita, JoanaBikov, AndrasMias, GeorgeBilichak, AndriyMichalak, MarcinBirgel, DanielMichalke, BernhardBlenner, MarkMichel, MaximilianBlombach, FabianMickol, Rebecca L.Bloomfield, SusanMiguel, González-PleiterBody, SimonMiki, YasuhiroBoeldt, Derek SMikolajewicz, NicholasBogan, ArthurMilano, SerenaBonfio, ClaudiaMilivojevic, VericaBorghini, AndreaMiller, William B.Borkowska, Edyta MartaMiranda, HugoBornemann, GerhildMiranda-Rius, JaumeBortner, Carl D.Mirete, SalvadorBos, Lieuwe D.Mirica, LiviuBoschetto, FrancescoMisaka, TomofumiBottari, TeresaMishra, MeerambikaBotton, ThomasMiśkiewicz, PiotrBouafif, HassineMitsuhiro, FukudaBougard, DaisyMiyamoto, ToshinobuBovee, Toine F.H.Mizuuchi, RyoBoyle, Kristen E.Mohamed, Azza H.Bozic, JoskoMojumdar, KamalikaBrack, AndreMok, Chee KengBradley, JohnMolina-Cerrillo, JavierBrady, AllysonMolina-Cruz, AlvaroBrągoszewski, PiotrMoniuszko, HannaBrasel, TrevorMonsarrat, PaulBraun, DieterMontecucco, FabrizioBrendan L., SkellyMonti, Maurilia MariaBrindisi, MatteoMoonen, XavierBrismar, TorkelMoran, JosephBrocco, EnricoMorandi, LucaBrodosi, LuciaMorel, Jean-LucBrogaard Larsen, JulieMoreno-Loshuertos, RaquelBroskey, Nicholas T.Mori, EmilianoBrunk, Clifford F.Moriyama, YuutaBrust, TarsisMorrison, IvanBuda, Valentina OanaMotamed, CyrusBuksińska-Lisik, MałgorzataMotiani, Kumail K.Bulfamante, Antonio MarioMotoki, TakakuBullock, EmmaMouga, TeresaBuonocore, FrancescoMoulin, AlexandreBurcynski, FrankMroczek, AgnieszkaBurgess, Steven J.Mrsa, VladimirBurke, RichardMucci, FedericoBurmann, BjörnMukaiyama, AtsushiBurton, Aaron S.Mulkidjanian, ArmenBurton, Zachary FMüller, LudmilaBussmann, Rainer W.Mullineaux, Conrad W.Byeon, HaewonMurab, SumitCacciottolo, MafaldaMuralidharan, SrideviCaetano-Anollés, GustavoMuratani, MasafumiCagnin, StefanoMuro-Pastor, Alicia M.Cai, Bi-HeMurphy, Patrick B.Caldwell, JacobMusalgaonkar, SharmishthaCalvo-Henriquez, ChristianMuzio, GiulianaCameron, JeffreyNadal, MiquelCampbell, Joshua WNadeau, JayCampoccia, DavideNaftalin, RichardCamprubi Casas, EloiNagy, ElodCañas, José A.Nagy, Janos B.Cannarella, RossellaNakai, KentaCanosa, StefanoNakajima, MasahiroCapanna, RodolfoNakamura, MasatoshiCapitanio, NazzarenoNakashiro, Koh-IchiCarapelli, AntonioNakayama, NaomiCardona, TanaiNakayasu, MasaruCarelli, StephanaNallasamy, PalanisamyCarlson, Jenna C.Namasivayam-MacDonald, AshwiniCarpentier, MathildeNamisaki, TadashiCarpi, SaraNannetti, GiulioCarraro, UgoNanno, MasanobuCarreras-Torres, RobertNarikawa, ReiCarugo, OlivieroNasiadek, MarzennaCaruso, GerardoNatarelli, LuciaCascella, RobertaNavarro, EstanisCasotti, RaffaellaNawrocki, GrzegorzCassier-Chauvat, CorinneNecas, TomasCastelnovo, MartinNegron-Ortiz, VivianCastresana, Javier S.Neigel, JosephCatalin, BogdanNelson, Fred R.T.Catanese, GaetanoNemoto, NaotoCauffiez, ChristelleNeunaber, ClaudiaCavedon, ValentinaNeveu, MarcCembrowska-Lech, DanutaNewman, Stuart A.Cercenelli, LauraNewton, MatildaCerri, JacopoNicolas, CyrilCéspedes, VanessaNiemiro, Grace M.Chai, Han-haNieri, PaolaChai-Adisaksopha, ChatreeNieto Feliner, GonzaloChan, Chia-HsinNieto Fontarigo, Juan JoseChan, ClementNieto, ManuelChang, Shin-TsuNikolaou, ElissavetChang, Wen-WeiNilsson, MariaChangey, FrederiqueNischwitz, Sebastian PChao, Chia-TerNishihara, HidenoriChapiro, JuliusNixon, Daniel W.Chatzitheodoridis, EliasNiyazov, DmitriyChatzopoulou, PaschalinaNolte, IngoChauhan, Madhulata SinghNorcic, GregorChen, ShujuanNoviello, CarmineChen, WeiyuNunes, Leonel Jorge RibeiroCheptsov, VladimirNunzio, VicarioCherepanoff, SvetlanaNürnberg, DennisChessa, Marco AdrianoNwosu, LilianChi, Wen-ChouOcampo Daza, DanielChigbu, De Gaulle IOchoa, JulioChilibeck, PhilOchsenkuehn, MichaelChiu, Hua-ShengOda, HirokiChiu, MartinaOey, MelanieChiu, Ya-HuiOgura, MasatsuneCho, Jin AhOhkawa, YukiChoi, MineeOhno, JunChoo, HyojungOkińczyc, PiotrChoromańska, BarbaraOlasagasti, FelixChristian, AlanOleksiewicz, UrszulaChudecka-Głaz, AnitaOliveira, Maria ConceiçãoCiampi, MarioOliver, OzohanicsCichero, ElenaOlivier, Jean LucCid, EmiliOlofsson, MalinCiobica, AlinOlszewska, MartaCirami, Calogero LinoOnisor, FlorinClanchy, FelixO’Reilly, ShaneClark, Benton C.Orlic, SandiClarke, PaulOrsi, William D.Cluley, VictoriaOsipova, ZinaidaCogo, SusannaOsterlind, KellCohen, Ana CarmenOtelea, DanCoimbra, SusanaOtręba, MichałColim, AnaOtt, Charlie MarkColla, EmanuelaOtto, SijbrenCollareta, AlbertoOvádi, JuditColonna, CristianaPage, AmandaComte, KatiaPagliara, Monica MariaConn, David BrucePal, SangitaConnolly Jr., Harold C.Pallottini, ValentinaContreras, AsunciónPalomo López, PatriciaCook, Jamie ElsilaPaloschi, ValentinaCooper, ElizabethPandey, SudhirCope, Kevin RichardPapalois, VassiliosCoppedè, FabioPardo, IsabelCoppey, MathieuPark, Jong-EunCordera, RenzoPark, Jun-BeomCordie, David R.Parodi, Pier CamilloCorenblit, DovParrozzani, RaffaeleCornelius, DenisePârvu, MarcelCosentino, GiuseppePasaje, Charisse Flerida A.Costa, Vera MarisaPascal, RobertCosta, VivianaPasman, Wilrike J.Coucha, MahaPatel, Jai SinghCoutinho, Maria FranciscaPathak, AshishCouvillion, SnehaPaton, ChrisCox, Cymon J.Paulo, AntónioCrisan, LuminitaPaulo, CarlosCrona, DanielPavia, MarcoCubiella, JoaquinPawlik, AndrzejCullimore, JuliePawlik, AnnaCuny, Gregory D.Pedrolli, DanielleCzechowski, Piotr OskarPeham, JohannesCzortek, PatrykPeixoto, LucianaDa Vià, MatteoPelagalli, AlessandraDabert, MiroslawaPellettieri, JasonDabrowski, FilipPelliccioni, PaoloDaca, AgnieszkaPeltekian, KevorkDaldello, Enrico MariaPeña-Llopis, SamuelDalla, EmilianoPeng, WenjingD’Angelo, LiviaPeng, YangDangwal, SeemaPeng, Yi-JenDayan, MichaelPereira, RuteDe Chiara, LetiziaPeriago, Maria VictoriaDe La Pena, MarcosPerkin, Lindsey C.De Leo, AntonioPerricone, CarloDe Martino, MarcoPeška, VratislavDe Palma, AnnaPetcherski, AntonDe Paz Fernández, Jose AntonioPetráš, MarekDe Rasmo, DomenicoPetrov, Anton S.De Riu, GiacomoPetsalaki, EvangeliaDe Sire, AlessandroPettersen, Veronika KuchařováDea-Ayuela, MaríaPezone, AntonioDearden, LauraPezzuto, AldoDeb, IndrajitPfeffer, Tobias JDebRoy, ChitritaPiano, IlariaDefez, RobertoPichrtova, MartinaDel Coso, JuanPiciu, DoinaDella Pepa, GiuseppePiedras, PedroDeme, PragneyaPieniazek, AnnaDerendorf, HartmutPietruszewska, WiolettaDeshmukh, RupeshPilic, LetaDettmer, UlfPing, XiaoliDevlieger, PatrickPintać, DiandraDi Bella, StefanoPinter, TylerDi Giulio, MassimoPipes, LenoreDi Gregorio, SalvatorePiscia, RobertaDi Marco, RobertoPivac, NelaDi Meo, IvanoPlasson, RaphaelDi Mise, AnnaritaPlendl, JohannaDi Rago, Jean-PaulPletser, VladimirDi Rosa, MichelinoPluth, Janice M.Di Rosa, RobertaPodgorski, Iva I.Di Sebastiano, KatiePodlewska, SabinaDi Stadio, AriannaPoeggeler, Burkhard H.Dias, Cristiano L.Polverino De Laureto, PatriziaDíaz Martínez, MargaritaPompili, SimonaDiéz-Pérez, IsmaelPond, DimityDing, Bao-JianPopova, Alexandra A.Djemiel, ChristophePorada, Christopher D.Do, Chi-WaiPoręba, RafałDoddapattar, PrakashPorpora, Maria GraziaDolgikh, Elena A.Porras, IgnacioDomínguez-Álvarez, EnriquePossik, EliteDomínguez-Martín, Maria AgustinaPoulet, LucieDominik, MartinPowers, Leanne CDominika, BednarczykPozzi, CarloDonahoe, MichaelPozzi, SamanthaDonat, Francisco Javier SilvestrePreiner, MartinaDonati, MassimilianoPrenafetaBoldú, Francesc XavierDong, Huan-JiPress, Adrian T.Donzelli, ElisabettaPreston, LouisaDorny, PierrePryzdial, EdwardDos Santos, Reinaldo SousaPrzybyłowski, TadeuszDraper, DavidPtaszkowski, KubaDresler, SławomirPugliese, NicolaDrozdovskaya, MariaPutzke, JairDuarte, LiliannePyatibratov, MichaelDubrova, YuriRabbow, ElkeDuca, FranzRachek, Lyudmila IDucza, EszterRadim, DobiášDujon, BernardRagnini-Wilson, AntonellaDupree, Jeffrey L.Rahimi, FaridDutcher, SusanRaimondi, LauraDuval, SimonRajesh, RamachandranDzhimak, StepanRalle, MartinaEbert, Andreas W.Ramesh, Sunita A.Ecke, ThorstenRamirez, RamsesEckert, Carrie A.Ramírez-Vélez, RobinsonEdwards, NicholasP.Ramkumar, VickramEfthimia, CotouRamos, CarmenEgel, RichardRampling, Michael WilliamEgli, MarcelRanjan, PeeyushEhira, ShigekiRantala, MarjaanaEkpenyong, Andrew EdetRavin, Nikolai V.Elber, RonReader, ArranEllestad, LauraRechowicz, KrzysztofElliott, BradleyRedmer, TorbenElmi, ChiaraReed, Karen RuthElsaid, Mohamed I.Reeksting, BiancaEmmett, BryanReitner, JoachimEmtestam, LennartRevel-Vilk, ShoshanaEngelberth, JurgenReverter Masia, JoaquínEngelhart, AaronReynolds, RichardEngevik, MelindaRezania, ShahabaldinEramo, StefanoRibaldone, DavideEric, MuirRibuffo, DiegoErtem, GözenRicagno, StefanoEscolà-Gil, Joan CarlesRicci, EmilianoEslam, MohammedRichter, ClaudiaEspinosa, JavierRicke-Hoch, MelanieEvguenieva-Hackenberg, ElenaRieck, ChristophEynard, Sonia E.Rigillo, GiovannaFahrenbach, AlbertRimmer, PaulFairen, AlbertoRiquelme, ErickFalzone, LucaRis, LaurenceFargašová, AgátaRishi, GautamFarnell, Morgan B.Rives, VicenteFehér, AttilaRizzarelli, EnricoFeilzer, AlbertRizzello, FrancescaFerla, MatteoRobben, JohanFernandes, BrunoRobin, Arif Hasan KhanFernández, José A.Robins, Jo Lynne W.Fernández-Mateos, M. PilarRobustelli, PaulFerri, FlaminiaRodrigues, Pedro M.Feulner, MartinRodrigues, RichardField, Daniel J.Rodriguez, AnnaFilacchione, GianricoRodríguez-Pérez, José ManuelFilippidou, SevastiRoggero, AngelaFink, DieterRoh, Yoon-SeokFiore, MicheleRomanenko, Svetlana A.Fisher, Caroline A.Rombolà, LauraFleming, CharlesRomeo, ClaudiaFlores García, EnriqueRoot-Bernstein, RobertFloria, MarianaRoques, Christian-FrançoisFonseca, LuisRose, Alan B.Fontaine, CoralieRose, GeorgForchhammer, KarlRoss, David S.Ford, NicoleRoss, Eric D.Forejt, JiříRota-Stabelli, OmarForhead, Alison J.Rovas, AlexandrosFornaro, TeresaRoy, DipankarForoncewicz, BartoszRoychowdhury, RajibForte, MaurizioRuan, YeChunFoulquier, FrancoisRückerl, DominikFrancis, FredericRueger, Maria AdeleFrank, AlexanderRuelland, EricFries, FabianRuf, AlexanderFujisawa, MasanoriRugani, PetraFujiwara, ShokoRugolo, MichelaFukuda, MakihaRuiz-Mirazo, KepaFukunaga, HisanoriRuonala, Mika O.Fukushima, KentaroRusso, GiorgioGać, PawełRusso, IsabellaGaire, BhaktaRuzzenente, BenedettaGalletti, FrancescoRyuzaki, SouGalli, RobertaSaad, Nizar Y.Galliciotti, GiovannaSack, Laura M.Gallo, GaetanoSaha, RanajayGalvez, JorgeSaha, Sushanta KumarGan, RenyouSahai, NitaGanatra, SarjuSaija, FranzGarab, GyozoSajdel-Sulkowska, ElizabethGarcía Iglesias, DanielSakamoto, TaiichiGarcia Sanchez, Angela M.Saladino, RaffaeleGarcía, YolandaSalehzadeh-Yazdi, AliGarcía-García, AndrésSalem, VictoriaGarcía-Ruiz, Juan ManuelSalerno, Alessandro GonzalezGarcia-Sherman, MelissaSalladini, EdoardoGartia, Manas RanjanSalman, MootazGary Alan, BassSalvi, NicolaGarzon, SimoneSalzano, GiovanniGassler, ThomasSamhan-Arias, AlejandroGavriilaki, EleniSampaio, Bruno LeiteGaza, RamonaSamuels, TobyGeisler, JonathanSanchez, OttoGemignani, FrancoSanchez-Martinez, MelchorGenovese, GiuseppaSantabarbara, StefanoGeorgiou, ChristosSantafé, ManelGeorgiou, DimitraSanthanam, AbiramiGhandhi, ShanazSantos, José MariaGherman, Calin MirceaSantos-Juanes, JorgeGhigo, AlessandraSantulli, GaetanoGhinassi, BarbaraSarecka-Hujar, BeataGhosh, PrarthanaSatake, HonooGianni, StefanoSato, HiroshiGiansanti, AndreaSauer, DanielGil-Calvo, MarinaSaurav, KumarGinaldi, LiaSawyer, AnneGinsburg, IdanSayers, Edward JGiordano, PaolaSazanov, LeonidGirard, ChristopheA.J.Sazonova, Margarita A.Girbardt, ChristianSchabussova, IrmaGladyshev, GeorgiSchiattarella, AntonioGoddard, AlanSchiattarella, GabrieleGoertzen, Leslie R.Schiavon, MarcoGolan, DanielSchick, EvanGomes, Carlos MartinsSchirmack, JanoschGómez Gómez, FelipeSchmidt, HannoGong, Sung SamSchrempf, DominikGonzález, JerónimoSchroeckh, VolkerGori, TommasoSchubotz, FlorenceGospodinov, AnastasSchulz, Benjamin L.Goswami, AnweshaSchulze-Makuch, DirkGoto, TaichiroSchunkert, HeribertGould, SvenSchwarz, RakefetGrabarek, Beniamin OskarScialò, CarloGrammatica, AlbertoScicali, RobertoGras, Laure-LiseScisciola, LuciaGraumann, Peter L.Scott, David A.Green, Nicholas JScuteri, DamianaGrefenstette, NatalieSeguí-Ripoll, José MiguelGrewe, FelixSekine, MasamiGrigorakis, KritonSekulic, DamirGrishkan, IsabellaSeligmann, HerveGrodzinsky, AlanSelvaraju, Ram KumarGroeneveld, DafnaSenel, MakbuleGross, Roxann DiezSequí-Canet, José MiguelGruić, IgorSerra, RaffaeleGu, LinxiaSerrano, JoseGuan, YutingSethi, GautamGuardado, IsmaelSeverinsen, Gro-HildeGuarnieri, MarcelloShamieh, SaidGudicha, Dereje W.Shang, QingsenGuerrero-casado, JoseShanmugam, MuruganandanGuerrieri, NicolettaSharma, VikramGugliandolo, EnricoSharma, ViragGunaje, JayaramaSharon, DeniseGupta, SushimShen, GuofangGurzu, SimonaShen, LongGutekunst, KirstinShenoy, PriyankGyulai, RollandSheppard, AdamHachisu, YoshimasaShi, HaifeiHadchouel, JulietteShi, XiaowenHagit, Baris-FeldmanShilev, StefanHammer, Bruce E.Shimizu-Inatsugi, RieHan, Yeon SooShin, Mal-SoonHannah, KaplanShinomiya, NariyoshiHansen, JohnShinozaki, YoshihitoHao, JihuaShmulevich, IlyaHarada, Bryan T.Shvarev, DmitryHarel, ItamarŠic Žlabur, JanaHaroun, RicardoSidelmann, Johannes JakobsenHarp, Joel M.Sieradzan, AdamHarrell, Carl RandallSilantiev, Vladimir VladimirovichHarry, WhitlowSilva, CatarinaHartman, MariuszSingh, ManvendraHattink, BartSingh, PradeepHatzipetros, TheoSingh, Vipul K.Hawkes, DavidSingla, BhupeshHayashi, Mariko KatoSinitsyn, Dmitry O.He, DaopingSjöqvist, ConnyHe, YongSkarzynski, Piotr HenrykHebb, Adam O.Skladany, LubomirHebbar, SaritaSkoura, LemoniaHeckmann, Bradlee L.Skuza, LidiaHeden, TimothySlivka, Dustin RusselHegarty, Shane V.Śliwiński, TomaszHeijink, IreneSmith, EricHellweg, ChristineSmreczak, MarcinHenriques, DoraSnead, ChristopherHerburger, KlausSnow, NicholasHerrero Moreno, AntoniaSolbiati, LuigiHervás-Marin, DavidSoler, David C.Hickman-Lewis, KeyronSolomon, LeeHihara, YukakoSolovyev, AndreyHillengass, JensSommer, FabianHillenkamp, JostSon, JongsangHirohata, SatoshiSong, YuyuHirota, Simon A.Sorci, GuglielmoHitka, MilošSoroko, MariaHoang, Nam V.Sorrentino, Nicolina CristinaHobbs, Russell P.Sorrentino, RosaHochgeschwender, UteSorsa, TimoHodges, RobertSoto-Hermida, AngelHoffmann, SigridSoumillion, PatriceHofmann, ElisabethSpinas, EnricoHolland, CeliaSponer, JuditHolm, Nils G.Springstein, Benjamin L.Holmstrom, Erik D.Squassina, AlessioHoney, Michelle Lorraine LewisSquillacioti, CaterinaHooker, Angelo B.Srivastava, SaurabhHorecka, BeataStanciu, LoredanaHorstkorte, RüdigerStavrinou, PinelopiHossain, Mohammad ZakirStefanyshyn, DarrenHough, KennethSteiger, MatthiasHouse, Christopher H.Stevens, Adam H.Howell, KevinStevens, DavidHrdy, JiriStewart, LucyHsiao, Sheng-MouStöllberger, ClaudiaHsieh, Tzung FuStrauss, SarahHsieh, Yi-ChenStrazewski, PeterHsieh, Yi-TingStrbak, OliverHua, XiaoqingStriker, Rob T.Huang, Cheng-WeiSu, Dong-MingHuang, HuangSuda, ShoichiroHuang, LanSuenaga, MitsukuniHuang, XiaobinSułowicz, SławomirHudgens, Jeffrey W.Summers, Katie LynnHuertas Romera, María JoséSun, LiHuljenić, DarkoSurguchov, AndreiHultdin, JohanSurleac, MariusHussain, Muhammad SajidSurman, AndrewHyett, JonathanSuryadevara, VidyaniIacolina, LauraSusuki, KeiichiroIchihashi, NorikazuSuzuki, MasaoIconomidou, VassilikiSvensson, Maria K.Icyuz, MertSymonová, RadkaIetto, GiuseppeSzebeni, Gábor JánosIglesias-Soler, EliseoSzewczyk, Nathaniel J.Ikehara, KenjiSzilagyi, RobertIles, GailSznajder, LukaszInan, Omer TTaanman, Jan-WillemIngram, Krista K.Tagami, TakayoshiInoue, KazushiTakafumi, OotsuboInoue, KeiichiTakahashi, RyuichiIosif, PediaditakisTakahashi, TakuIovino, DomenicoTakai, KazuyukiIshorst, NinaTallent, JamieIsola, GaetanoTalukdar, SarmisthaIsozaki, TakeoTamura, KojiIspoglou, TheocharisTan, Bee KIvanovska, NinaTanaka, TakujiIwaya, YugoTang, FengRuIżykowska, KatarzynaTaoro-González, LucasJablonowski, ZbigniewTarakhovskaya, ElenaJacint, TokolyiTasca, GiorgioJadoul, MichelTegl, GregorJähn, KatharinaTeisseyre, AndrzejJain, Shri MohanTekbas, AysunJampilek, JosefTellez-Isaias, GuillermoJang, Suk YongTeo, JeremyJangampalli Adi, PradeepkiranTerauchi, KazukiJankovská, IvanaTerentyev, Vasily V.Jankowska, MilenaTesarik, JanJankowski, MarekTessaro, FrancescaJanz, DavidTessera, MarcJardine, MarieTetaud, EmmanuelJarome, TimothyThamatrakoln, KimberleeJavaheri, TaherehThanigaimani, ShivshankarJavid, MahsaThiel, TeresaJensen, Rikke BeckThomas, John C.Jeong, Dong-HoonThompson, JulieJeong, SekyooTilocca, BrunoJerez-Cepa, IsmaelTocmo, RestitutoJheeta, SohanTodt, DanielJia, TonyTogawa, AtsushiJiang, Rong-SanTomita-Yokotani, KaoriJimenez-Perez, IreneTompkins, Thomas A.Jin, EonSeonTorregrosa, RubenJin, HongTosolini, AndrewJoers, ValerieToto, AngeloJohnson, XenieTõugu, VelloJohnston, J. SpencerTrajkcovic, NebojsaJonathan, ElliottTrakada, GeorgiaJones, D. DafyddTraustadόttir, TinnaJordan, SeanTrembath-Reichert, ElizabethJørgensen, Anders PalmstrømTrigo-Rodríguez, Josep M.Juškaitis, RimvydasTripathi, VishnuKabani, MehdiTripette, JulienKabeya, NaokiTrosko, JamesKaczynski, PiotrTsai, Cheng-HsiuKaddour, HusseinTsay, Shwu-ChenKagami, HideakiTsika, ElpidaKalani, KomalTsui, Ke-hungKaludercic, NinaTsui, Kuan HaoKam, AntonyTsuji, KenjiKamennaya, NinaTung, Tao-HsinKandeel, MahmoudTurdo, AliceKanitakis, JeanTürke, AndreasKar, SouvikTyson, JulianKaradjian, GregoryTyystjärvi, TainaKarakasiliotis, IoannisUmehara, TakuyaKarev, GeorgyUrbanek-Trzeciak, Martyna O.Karolkiewicz, JoannaVallée, YannickKarouia, FathiVan Dijl, Jan MaartenKataoka, YukiVan Haren, Simon D.Kato, YusukeVan Krieken, RichardKaushik, Nagendra KumarVan Noorden, Cornelis Johannes ForrindinisKawano, RyujiVandepas, LaurenKawasaki, KiyonoriVanhie, ArneKazmierczak, JozefVarrella, StefanoKebukawa, YokoVarsi, CecilieKelarakis, AntoniosVaz, CátiaKempsell, Karen EVerbeek, D.S.Kenawy, NihalVerni, MichelaKeren, NirVerstegen, Ruud H.J.Kereszturi, AkosVidal-Meireles, AndréKesten, ChristopherVijverberg, KittyKevern, PeterVilla, AnnaKhalf, AbdurizzaghVilla, ChiaraKhatri, VishalVilović, MarinoKhundmiri, Syed J.Vincent, Jean-louisKhung, Yit LungVinn, OlevKido, TatsuoVinogradoff, VassilissaKilanowicz, AnnaVisscher, AnneKim, BongleeViswakarma, NavinKim, Chang-BaeVita, RobertoKim, Dong HyunVitek, PetrKim, Gi JinVitiello, LivioKim, Ho YeonVolny, MichaelKim, Jae SeokVrečer, FrancKim, LouWade, Rachael MKim, SunghyunWaghmare, SakharamKim, Young JunWagner, Erwin F.Kitadai, NorioWagner, PeterKitagawa, JunichiWaidmann, SaschaKitagawa, SeiichiWaithman, JasonKladnik, AlešWajima, TakeakiKlähn, StephanWalczewska, AnnaKlegerman, Melvin E.Walf, Alicia A.Klimontov, VadimWalters, GilesKnobe, MatthiasWang, AnnaKobashi, MotoiWang, Mong-HengKobayashi, KenseiWang, SishuoKoch, MoritzWang, Tsung-JenKoczyk, GrzegorzWanner, SamuelKoehler, KarstenWard, Christopher SKoh, JinWard, Rachelle M.Koh, Jin-HoWarke, MatthewKohlhof, HendrikWassef, MichelKojima-Yuasa, AkikoWatts, StephanieKolb, VeraWatzer, BjörnKolbe, DianaWawrzkiewicz, MonikaKoll, DanielWei, JinKong, LingboWeiergräber, MarcoKonieczka, PawełWeigt, DorotaKonôpková, AlenaWeimer, Louis H.Kopacz, AleksandraWeinzierl, RobertKoppel, RossWeitzendorfer, MichaelKordyum, ElizabethWeng, XiaoyuKorpelainen, HelenaWes, ZimmermannKosan, ChristianWestenskow, PeterKoshev, Yordan S.Wester, MichaelKosinska-Kaczynska, KatarzynaWideman, JeremyKosinsky, Robyn LauraWieczerzak, EwaKostrzewa, Richard M.Wieczorek, KingaKotsyurbenko, OlegWilczynski, MiloszKouokam, J. CalvinWilde, AnnegretKouw, ImreWilliams, GavinKouzu, HidemichiWitzany, GuentherKovács, BélaWong, AngelKovalski, JoannaWong, Chin-CheanKoyama, YuWong, JarodKrabbenhoft, Trevor J.Woo, So-YounKrasińska, BeataWoo, Sun HeeKremer, DavidWood, BenKretzschmar, KaiWood, Claire LKries, HajoWrześniok, DorotaKrijgsheld, PaulineWu, HongluKroneck, PeterWu, Meng-YuKronenberg, AmyWu, Ting-FengKrüger, MarcusWyatt, SarahKrulová, MagdalénaXavier, Joana C.Krupa, RenataXie, MouzheKrzyżanowska-Kowalczyk, JustynaXie, YuweiKubica, PawełXystrakis, FotiosKukreja, SubhashYadav, SuryaKular, LaraYahia, MassinissaKumar, DhirendraYakovlev, VasilyKumar, GauravYamagata, YoshiyukiKumar, RavindraYamamoto, TatsuyukiKumar, ShaileshYamaoka, YusukeKume, KensukeYanaka, NoriyukiKun, ÁdámYang, Cheng-YaoKurtenbach, StefanYang, RuiguoKurz, KatharinaYang, Woong MoKusuhara, SentaroYen, KelvinKuter, KatarzynaYi, MyunggiKwiatkowski, SebastianYoder, KristineL.Kaufman, HowardYoshida, MikaLa Favor, JustinYoshihiro, FurukawaLa Mendola, DiegoYu, HeLa Tessa, ChiaraYu, JeongilLabenz, ChristianYu, Sheng-ShengLacasta, AnnaYun, ChawonLacina, LukasZachou, KalliopiLadoukakis, ManolisZaitlin, DavidLafon, DavidZając, MirosławLafon, MoniqueZamyatnin, AndreyLaganà, Antonio SimoneZancanaro, CarloLambova, SevdalinaZanden, Crystal VanderLambrev, PetarZandi, RoyaLambreva, Maya D.Zawal, AndrzejLamparter, TilmanZdzisińska, BarbaraLandeck, NatalieZedler, Julie A. Z.Landi, ClaudiaZelenik, KarolLanglois, DanielZeng, ShuwenLark, DanielZhang, DianbaoLatifi, AmelZhang, LiLebedev, VadimZhang, XinLeclercq, IsabelleZhang, YeLeclère, ValerieZhang, ZhaoleiLederkremer, Gerardo Z.Zhao, YuLee, Chien-TeZheng, XiangjianLee, HeedooZhou, ChiLee, Jiwon M.Zhou, MingqiLee, WanhyungZilberg, DinaLee, YureeZimmer, MarcLefevre, Sophie D.Zimmermann, AndreasLegrand, SylvainZmarzły, NikolaLehmer, OwenZmonarski, SławomirLelešius, RaimundasZozulia, OleksiiLevi, MosheZupańska, Agata KLevolger, StefZyśk, MarlenaLi, Guannan

